# Responding to COVID-19 on the outer islands of Tuvalu

**DOI:** 10.5365/wpsar.2024.15.2.1080

**Published:** 2024-05-09

**Authors:** Karen Hammad, Lily Tangisia Faavae, Aloima Taufilo, Margaret Leong, Viliame Nasila

**Affiliations:** a Menzies Health Institute Queensland, Griffith University, Gold Coast, Queensland, Australia.; b College of Nursing and Health Sciences, Flinders University, Adelaide, South Australia, Australia.; c Division of Pacific Support, World Health Organization, Suva, Fiji.; d Ministry of Social Welfare and Gender Affairs, Funafuti, Tuvalu.; e Fiji Emergency Medical Assistance Team, Ministry of Health and Medical Services, Suva, Fiji.; f Pacific Community, Suva, Fiji.

## Abstract

**Problem:**

Coronavirus disease (COVID-19) reached Tuvalu’s shores in November 2022, making Tuvalu one of the last countries in the world to experience community transmission of the disease. With minimal capacity to deliver critical care and a small health workforce that had been further depleted by COVID-19 infection, response priorities rapidly shifted to the outer islands.

**Context:**

The outer islands are accessible only by boat, with travel taking from 6 to 24 hours. The return of high school students to their home islands for the Christmas holidays had the potential to place further pressure on the islands’ medical facilities.

**Action:**

A multiorganizational collaboration between the Australian and Fijian governments, the Pacific Community, the Tuvalu Ministry of Social Welfare and Gender Affairs (MoHSWGA) and the World Health Organization facilitated the deployment of two teams to the outer islands to provide support.

**Outcome:**

The team worked with public health and clinical staff to provide technical support for clinical management, infection prevention and control, laboratory, risk communication, community engagement and logistics.

**Discussion:**

The outer islands’ response to the pandemic significantly benefited the island communities, the MoHSWGA and the team members who deployed. The key lessons identified relate to the need to strengthen the health workforce and supply chain.

## PROBLEM

In November 2022, Tuvalu became one of the last countries in the world to experience an outbreak of coronavirus disease (COVID-19) when community transmission was detected on the main island of Funafuti. (*1*) With a land mass of 26 km^2^ consisting of Funafuti and eight outer islands (OIs), Tuvalu is one of the smallest and most remote countries in the world. (*2*) Approximately 40% of Tuvalu’s population of 11 000 live on the OIs. (*2*) In March 2020, with the primary objective of a COVID-19-free country, the Government of Tuvalu declared a state of emergency and initiated strict border measures. (*3*) As a precautionary measure to protect against the transmission of the virus to the OIs, the Government mandated the relocation of people from Funafuti back to their home islands and prohibited people returning to Funafuti. (*3*, *4*) This led to a 35% increase in the OI population. (*5*)

Shortly after a COVID-19 outbreak was reported in Funafuti, the OI of Nui detected COVID-19 in a traveller returning from Funafuti. This coincided with the end of the school year and the imminent return of nearly 500 boarding school students to their home islands. The possibility that the return of students could induce community transmission of COVID-19 across all OIs caused concern as health resources were likely to be rapidly overwhelmed. Estimations at the time suggested that a high percentage of the OI population had risk factors for severe COVID-19 disease, such as people aged over 60 years and those diagnosed with one or more noncommunicable diseases, in addition to pregnancy or smoking. (*6*)

Of particular concern was the potential for those with risk factors to develop severe or critical COVID-19 disease and requiring higher levels of treatment not available on the OIs. This prompted Tuvalu’s Permanent Secretary for Health to make a formal request to the World Health Organization (WHO) for technical support. On 19 November 2022, Fiji, specialized agencies of the United Nations and partners deployed a chartered flight with medical supplies and health experts to Tuvalu. (*1*)

## CONTEXT

Travel to the OIs and atolls from Funafuti takes at least 6 hours to reach the closest islands and up to 24 hours to reach Nanumea, the northernmost island. Travel to the OIs is only by government-run boat, which is costly and sometimes treacherous, resulting in injuries and fatalities when seas are rough. During the pandemic, the boat schedule was maintained so that fuel, food and medical supplies could be transported to the OIs. Each island imposed its own strict requirements such as pre-departure rapid assessment testing of passengers and crew, followed by quarantine measures on arrival. Some of the islands’ authorities also imposed other travel restrictions such as not allowing people other than their own residents and health professionals to come to their islands.

The OIs have limited health resources. Apart from Vaitupu and Niulakita, all have just one health clinic served by a small team of health personnel, in most cases comprising a nurse, nurse’s aide and sanitation officer. (*2*) Vaitupu, which hosts the only government-run high school in the country, has two clinics, one of which serves the transient high school population, which reaches 500 pupils during school terms. Currently, there is no purpose-built clinic on the island of Niulakita and the local nurse uses her own house as a makeshift clinic to serve a population of around 40. Critically unwell patients on OIs are reliant on transport to Funafuti by boat to Princess Margaret Hospital (PMH). The only hospital in Tuvalu, PMH is a 50-bed facility that provides primary- and secondary-level care and limited diagnostic services. (*2*, *3*) A significant amount of the health expenditure is spent on the Tuvalu Medical Treatment Scheme, whereby Tuvaluans are sent overseas to access specialty health care. (*2*)

## ACTION

Multiorganizational collaboration between the Australian and Fijian governments, the Pacific Community, the MoHSWGA and WHO facilitated the deployment of two teams to the OIs to provide technical support. (*1*) Team members had expertise in care pathways and clinical management, environmental health, hospital management, infection prevention and control (IPC), laboratory and biomedicine, public health, risk communication and community engagement. Local doctors and nurses from the MoHSWGA travelled with the teams to strengthen the workforce on the OIs, which had been depleted due to COVID-19 infections.

**Fig. 1:**
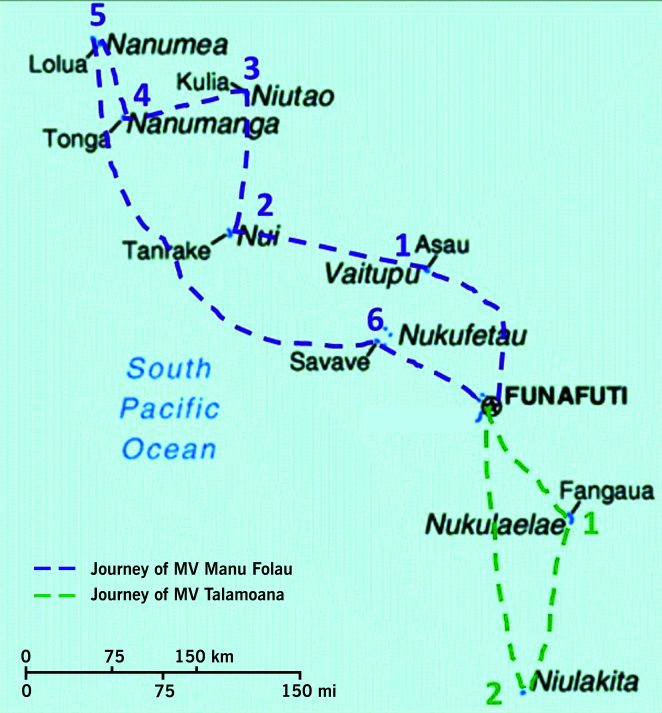
Routes of two deployment teams on Marine Vessel (MV) Manu Folau and MV Talamoana from Funafuti to the outer islands, December 2022

The first team who left Funafuti on the Marine Vessel (MV) Manu Folau on 4 December 2022 travelled for 6 days to the north and central islands (**Fig. 1**). A second team departed Funafuti on 13 December 2022, travelling by MV Talamoana on a 3-day journey to the southern islands of Nukulaelae and Niulakita (**Fig. 1**). The teams brought medical equipment, personal protective equipment (PPE), essential medicines and COVID-19 therapeutics, laboratory equipment and larger items such as hospital beds, oxygen concentrators, autoclaves and large oxygen tanks as well as fresh drinking-water, as many of the islands were experiencing drought.

[**insert** Figure 1]

On each island, the teams spent considerable time at the health clinics redirecting and strengthening care pathways by providing technical assistance and refresher trainings in IPC, oxygen escalation, therapeutic management and clinical management; developing physical spaces for emergency management of severe and critical cases; performing maintenance and repair work on medical equipment; and providing clinical care such as treating outpatients and assisting with decision-making for complex cases. Team members also escorted local staff on home visits or mobile testing on some of the islands.

## OUTCOME

By the time the teams arrived in December 2022, all the islands had reported COVID-19 cases but were at different stages in their response and had different COVID-19 measures in place. Most cases presenting to the clinics were non-severe, requiring outpatient treatment with medications for symptom relief such as paracetamol and ibuprofen or testing to confirm their COVID-19 status. Although manageable, this increased the workload at the clinics. The few critically ill patients (COVID-19 or non-COVID-19) were discussed with specialists on Funafuti and were medically evacuated by boat if deemed necessary.

Notable on all islands was the effect of drought, for which Tuvalu had declared a state of emergency on 8 November 2022. (*7*) The effects of drought were exacerbated on some islands where the desalination plants had malfunctioned. It was difficult to get the desalination plants fixed earlier due to the combined effects of geographical isolation and supply chain disruptions during COVID-19. All islands had broken or missing equipment important for health-care delivery such as thermometers, oxygen cylinders, electrocardiograph machines and sphygmomanometers (blood pressure monitors). In addition, some of the building infrastructure was in need of repair, such as air conditioning units and autoclaves.

As well as provisioning the islands with essential medicines, equipment and supplies, the deployment provided the health staff with face-to-face training in clinical care, IPC and sample collection. This training is rarely conducted due to the islands’ isolation and distance from Funafuti and, more recently, due to the COVID-19 pandemic restrictions. Defective equipment was repaired, physical spaces were rearranged to optimize clinical care, and gaps were identified and reported to the MoHSWGA for action.

For the visiting teams, the journey to the islands was not without incident, with many team members experiencing seasickness particularly on the longer journeys. The MV Talamoana developed a fuel leak and was stranded for 12 hours until the engineers could fix it. Also, on that journey, it was not feasible to disembark on Niulakita due to rough seas. Instead, the community sent two members in a small vessel from the island to collect equipment and supplies from MV Talamoana and transfer a patient who needed to return to Funafuti.

## Discussion

Geographical isolation and supply chain disruptions presented a challenge for health workers who did not always have access to items such as PPE, testing kits, therapeutics or oxygen. They had to manage with broken, malfunctioning or missing equipment and infrastructure. The absence of alcohol-based hand rubs for hand hygiene on some OIs, combined with malfunctioning desalination plants and drought, had clear implications for effective IPC, making it difficult for health clinic staff and patients to adhere to hand hygiene principles.

Supply chain disruptions impacted many Pacific island countries and areas (PICs) during the COVID-19 pandemic, leading to an increase in the cost of products and difficulties in obtaining essential medical supplies. (*8*, *9*) The effect of COVID-19 on supply chains globally has been well reported, with the pandemic highlighting global supply chain vulnerabilities related to critical medical supplies. (*9*-*11*) This supports the implementation of strategies such as the pre-positioning of essential supplies to ensure that they reach remote communities and that access can be maintained during times of crisis.

During the outbreak, an increase in health clinic presentations placed pressure on the small OI workforce, which was not prepared to manage the large influx of patients. It was fortunate that very few people on the OIs required higher-level care as health workers on the OIs were not well equipped to manage critical patients. Currently, the Government of Tuvalu is building airstrips on each OI, which may reduce their geographical isolation and ensure that critically unwell patients have faster access to higher levels of care. Lessons from this deployment emphasize that this project should be combined with strengthening maintenance schedules and supply chains for essential equipment and supplies, as well as providing appropriate training for health workers to utilize equipment safely and effectively. The need to strengthen critical care in the region was identified at the beginning of the COVID-19 pandemic, and nurses in many PICs, including Tuvalu, were upskilled in critical care. (*12*)

The deployment to the OIs was beneficial in terms of ensuring that technical support and medical supplies were delivered during a time when the OIs were experiencing uncertainty. The deploying teams also reassured OI communities by supporting and ensuring that health workers on the OIs were included in the MoHSWGA national COVID-19 response. The strengthening of care pathways and clinical processes, as well as the visiting teams’ sharing of technical knowledge and skills with the local staff, helped improve confidence among local health workers. Upon their return, the teams were also debriefed by the MoHSWGA on the needs, strengths and gaps of each island. This was a unique opportunity for the international members of the deploying teams to exchange knowledge, as the OIs of Tuvalu are rarely visited by outsiders. The deployment also provided an opportunity for other team members with ties to the OIs to visit their friends and families and give back to their own communities.

This deployment is a good example of how regional and international cooperation has strengthened the networking and collaboration capacity of senior administrators in the MoHSWGA in preparation for future health emergencies. The lessons outlined in this paper provide points for consideration when preparing for future outbreaks in remote OIs or PICs. This experience highlighted two key areas of focus for future pandemic or outbreak preparedness for the OIs, namely, access to essential medical supplies during times of crisis and capacity strengthening to manage critically unwell patients in the OIs.
